# Inequalities in Trust Levels and Compliance With Physical Distancing During COVID-19 Outbreaks: Comparing the Arab Minority and Jewish Populations in Israel

**DOI:** 10.3389/ijph.2022.1604533

**Published:** 2022-04-05

**Authors:** Haneen Shibli, Daiana Palkin, Limor Aharonson-Daniel, Nadav Davidovitch, Nihaya Daoud

**Affiliations:** ^1^ School of Public Health, Faculty of Health Sciences, Ben-Gurion University of the Negev, Negev, Israel; ^2^ PREPARED Center for Emergency Response Research, Negev, Israel; ^3^ Department of Public Health, School of Public Health, Faculty of Health Sciences, Ben-Gurion University of the Negev, Negev, Israel; ^4^ Department of Health Systems Management, School of Public Health, Faculty of Health Sciences, Ben-Gurion University of the Negev, Negev, Israel

**Keywords:** COVID-19, compliance, trust in information, physical distancing, minorities

## Abstract

**Objectives:** This study explores associations between trust in directives and compliance with physical distancing by comparing two populations in Israel.

**Methods:** Following two lockdowns, we conducted two cross-sectional surveys among the Arab minority and Jewish citizens of Israel (first survey, *N* = 613; second survey, *N* = 542). We conducted multivariable logistic regression analyses for the association between trust and compliance with physical distancing separately for each group in each survey.

**Results:** In both surveys trust levels were significantly lower among Arabs than Jews (*p* < 0.001). Compared to Jews, Arabs were less likely to report compliance with physical distancing in the first and second surveys (OR = 0.52, 95% CI 0.32–0.84 and OR = 0.62, 95% CI 0.39–0.98, respectively). In both surveys trusting the directives was an important determinant of compliance with physical distancing among Jews only.

**Conclusion:** Our findings indicate that momentum is important in building and maintaining public trust and compliance during pandemics. Policymakers should note the lack of trust among Arabs, which warrants further research and interventions.

## Introduction

Israel reported its first case of COVID-19 on 21 February 2020 (1). Weeks later, after the declaration by the World Health Organization (WHO) of a global COVID-19 pandemic on 11 March 2020 (2), the country introduced guidelines for the public on physical distancing, hand washing, and mask-wearing (3). The Israeli government took multiple measures to reduce social interactions and increase physical distancing. As of 19 March 2020, Israel’s Prime Minister declared a national state of emergency and, soon afterward, the government approved emergency regulations, including closures of schools, universities and shopping malls, limitations on activities in the public and private sectors, restrictions on faith institutions, and lockdowns in several neighborhoods and cities (4). Besides the challenges of physical distancing, the COVID-19 pandemic introduced tremendous uncertainty into people’s lives. People were inundated with an “infodemic” of news from multiple outlets, sometimes with contradictory messages. As a result, it was difficult for people to determine the trustworthiness of informational sources (5).

People’s understanding of information and their willingness to act based on it are influenced by their trust in the information sources (6). In China, risk perceptions of COVID-19 varied depending on whether the information came from social media or mass media (7). While private media sources distribute messages that can reduce public trust in scientific knowledge and health policies, other sources may have the opposite effect (8). For example, research conducted among Canadian citizens found that social media use was associated with more misperceptions about COVID-19 and less compliance with social distancing measures (9). Researchers in the United States reported that trust in government information sources during COVID-19 was positively associated with adherence to physical distancing guidelines, and these sources were also regarded as the most trusted ones (10). Additionally, extensive research in several European regions demonstrated that, during the COVID-19 pandemic, there was significantly greater compliance with health policies and restrictions by individuals who had more trust in the government (11).

Trust and the behavior of the public vary (12). We cannot understand the relationship between trust and compliance with physical distancing during the pandemic without accounting for the characteristics of various population groups. Previous studies have shown that, in general, ethnic minority groups have little trust in government and government decision-makers (13), healthcare systems (14, 15), health policy setters (16), and physicians (17) compared to other majority ethnic groups. Moreover, ethnic minority groups such as African Americans in the United States and Black and South Asian communities in the United Kingdom were disproportionally affected by the COVID-19 pandemic (18–20).

As of 2021, Israel’s population is over 9 million. About 74% are Jews of all backgrounds, and 21% (or nearly 1.9 million) are Palestinian Arabs (hereafter Arabs) (21), who comprise the country’s largest ethno-national minority. The Arabs are an indigenous minority that has been suffering from structural discrimination for many decades (22). They have low socioeconomic status (23), limited access to healthcare services (24, 25) and low levels of health literacy (26). These factors may make them more vulnerable to serious health consequences from COVID-19, which might also reduce their trust (22, 27). Moreover, the Arab minority has a greater prevalence of chronic diseases and other risk factors such as smoking and obesity that exacerbate COVID-19 (28, 29).

Given this background, we explored the association between trust in information about the pandemic and compliance with physical distancing regulations among the Arab minority and Jewish majority populations in Israel.

## Methods

### Study’s Design and Setting

The Ethics Review Board of the Faculty of Health Sciences at the University approved our study. We conducted two cross-sectional surveys of Jewish and Arab adults (≥18 years old) residing in Israel during the COVID-19 outbreak following Israel’s first (April–June 2020) and second (October–November 2020) lockdowns (30). A convenience sample was obtained *via* an online structured questionnaire using Qualtrics software via a hyperlink distributed through social media in Arabic and Hebrew. While the use of online survey platforms has various limitations, this technology was most suitable for collecting data while maintaining physical distancing during the pandemic. The online questionnaire included questions regarding socio-demographics, state anxiety, physical health and health behaviors, COVID-19 related information, and compliance with physical distancing in the participants’ environment. The last section asked about trust in COVID-19 information sources and state anxiety.

### Measures

The dependent variable compliance with physical distancing was assessed by the question: “To what extent do you feel that people in your environment comply with physical distancing to prevent the spread of the coronavirus?” We asked about physical distancing in the participants’ environment to avoid social desirability bias. In Israel, failure to maintain physical distancing violated the emergency public health law, and people who did so could face legal sanction. The answer was scored on a 5-point Likert scale, with a range of (5) always comply to (1) never comply. This variable was dichotomized at the median score (=3) into high (3 < median) and low (3 ≥ median) levels of compliance with physical distancing.

The main independent variable, level of trust in the directives, was measured by nine questions regarding trust in the sources of information (Israel’s Prime Minister, the Ministry of Health, Magen David Adom (National Emergency Forces), the country’s national emergency medical services (EMS), health maintenance organizations (HMOs), local authorities, social media, a family physician or nurse, and local associations or professional committees. Respondents indicated their level of trust on a 5-point Likert scale ranging from (1) not at all trusted to (5) very much trusted. We calculated the mean score for each information source and then created a scale of trust from the means of the nine items. The median of the total score was 3. The total scale of trust was then dichotomized into high (3 ≥ median) and low (3 < median) trust levels. The reliability test revealed a Cronbach’s alpha of 0.69 for Jewish participants and 0.71 for Arab participants.

Independent variables included:

(1) Socio-demographics:
  • Ethnicity was determined by participants’ self-reported ethnic identity as “Jewish” or “Arab”.
  • Age as a continuous variable was assessed by year of birth and was categorized into five groups (18–29, 30–39, 40–49, 50–59 and 60+).
  • Gender was categorized as male or female.
  • Marital status was assessed by asking if the participants were single, married, single parent, divorced or widowed. The variable was dichotomized into two categories: married and other.
  • Religiosity level was categorized into three categories: not religious, traditional and religious.
  • Education level included five categories (no formal education, elementary school, partial high school, full high school and academic degree) that were dichotomized into two categories: up to high school and academic degree.
  • Relative income included five categories (much less than average, less than average, similar to average, more than average or much more than average) that were categorized into three categories: below average, same as average and above average.
  • History of chronic disease was grouped into two categories (yes/no).
  • Country of birth was grouped into two categories: Israel and other.
  • Smoking was grouped into two categories (yes/no).
(2) State anxiety was assessed by Spielberger’s State-Trait Anxiety Inventory (STPI) (31) based on a 5-point Likert scale. We computed the mean score of answers to create a total score of scale anxiety that we dichotomized by the median as follows: low anxiety (2.90 < median) vs. high anxiety (2.90 ≥ median). Cronbach’s alpha was 0.91 for Jewish participants, 0.89 for Arab participants.
(3) Provision of general COVID-19 information in the native language was assessed using a question: “To what extent do you feel that COVID-19 information has been provided in your native language by the bodies responsible for COVID-19 crisis management?” Answers were categorized as slightly, moderately and very much.
(4) Change in employment status due to the spread of COVID-19 was assessed using the question: “Have you lost your job or have your working hours been reduced due to the spread of the coronavirus?” The answers were categorized into yes, no/other, and not clear yet.
(5) Refraining from seeking healthcare was assessed using the question: “Have you refrained from using healthcare services when you needed them during the past 6 months?” Answers were dichotomized into yes/no responses.
(6) Familiarity with people who died from COVID-19 was measured by: “Do you know people who died from COVID-19?” Answers were dichotomized into yes/no responses.

### Statistical Analysis

Data analysis was conducted using IBM’s Statistical Package for Social Sciences (version 25.0). There were no missing data, as respondents had to complete all mandatory questions. After examining the data and calculating the different variables, we identified the descriptive statistics. In the univariate analysis, we used the Chi-square test for categorical variables. Statistical significance was set at *p* < 0.05 for the analysis. Spearman’s correlations were calculated to avoid multicollinearity. No correlations were found beyond the threshold correlation coefficient (r) of 0.7. Based on the univariate findings, several multivariate logistic regression models were used to estimate the odds ratio for the associations between trust and compliance with physical distancing, while adjusting for the other independent variables associated with compliance in the univariate analysis (*p* < 0.05). Before conducting the logistic regression, we examined the confounding effects and interactions of the independent variables on the main association. The logistic regression models were for the total sample and separately for each ethno-national group.

Multivariate regression models in the first and second surveys included the same models and were adjusted for the same variables except for different interactions that were included in the different surveys. The models in the logistic regression were as follows. Model 1 was unadjusted and estimated the association between trust in information sources and compliance with physical distancing. Model 2 was adjusted for level of trust, age groups, ethnicity, state anxiety level, religiosity level, relative income, marital status, education level, and information provided in the native language. Model 3 was adjusted for the variables in model 2 and the interaction between marital status and relative income. In the second survey, the same set of variables was included in the first and second models, but in model 3 we adjusted for the variables in model 2 and for the interaction that was found between religiosity level and information provided in the native language.

## Results

In total, 613 participants completed the first online survey, of whom 332 (54%) were Jewish and 281 (46%) were Arab. The second survey included 542 participants, 319 (59%) Jewish and 223 (41%) Arab.

**FIGURE 1:**
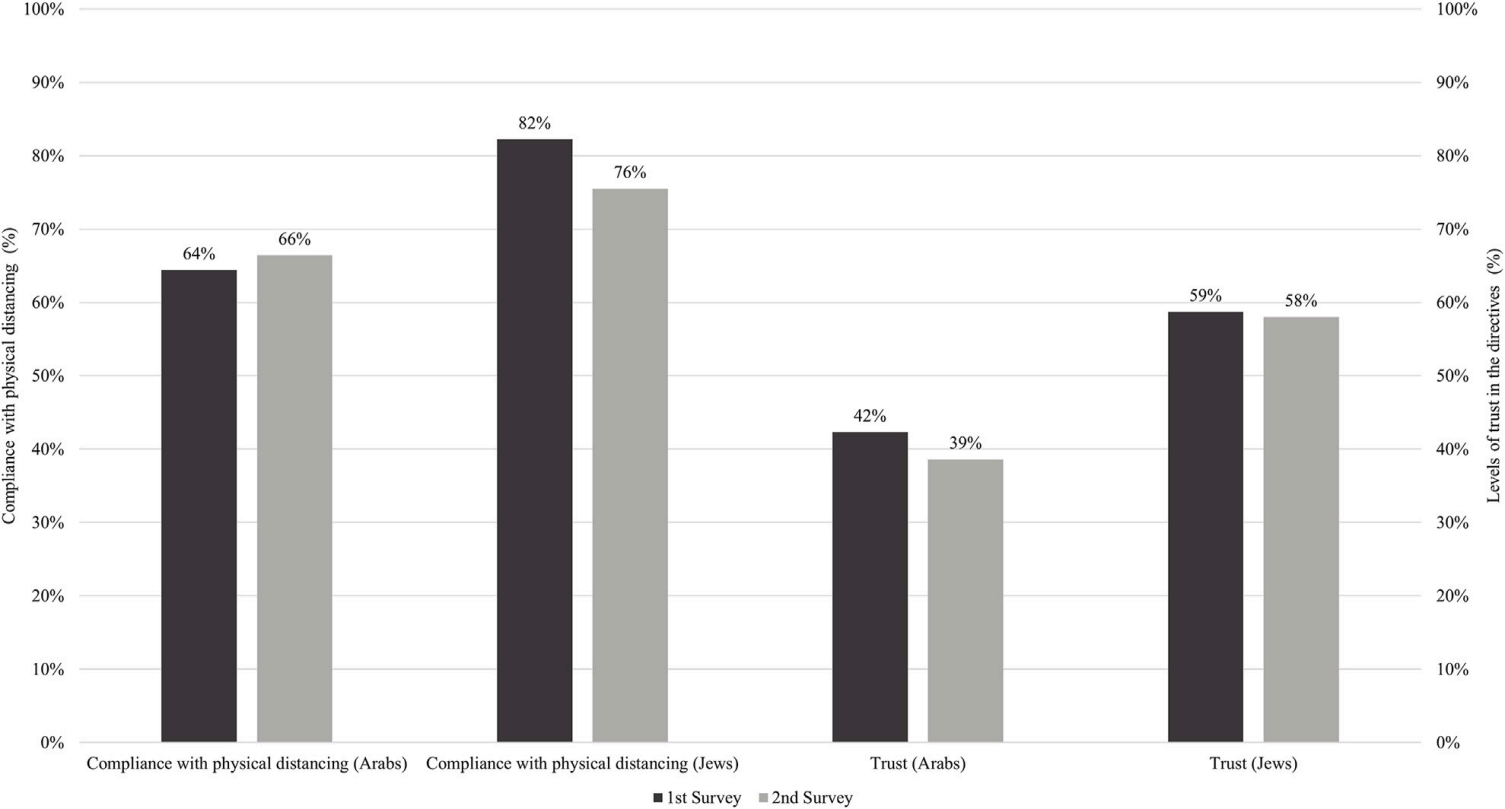
Compliance with physical distancing and levels of trust in the directives among Arab and Jewish participants in the first (*n* = 613) second (*n* = 542) surveys (Israel, 2020). Inequalities in Trust Levels and Compliance With Physical Distancing During COVID-19 Outbreaks: Comparing the Arab Minority and Jewish Populations in Israel (Israel, 2020).

Figure 1 illustrates significant differences between Jewish and Arab participants in compliance with physical distancing in both surveys. In the first survey, significant differences (*p* < 0.001) were observed in compliance with physical distancing among Jewish participants (82%) compared to Arab participants (64%). Significant differences (*p* < 0.05) were also observed in the second survey, as 76% of the Jewish participants reported compliance with physical distancing compared to 66% of the Arab participants. Figure 1 also illustrates significant differences (*p* < 0.001) between Jewish and Arab participants in the level of trust in information sources in both surveys. In the first survey, 59% of the Jewish participants reported a high level of trust compared to 42% of the Arab participants. Significant differences (*p* < 0.001) were also observed in the second survey, as 58% of the Jewish participants reported a high level of trust compared to 39% of the Arab participants.

There were differences between the two groups in the first survey about the trust in each information source. The Arab participants had significantly (*p* < 0.05) less trust than the Jewish participants in information from Israel’s Prime Minister, MDA, HMOs, the local authority in their locality, social media, and their family physician or nurse. In the second survey, these differences persisted except for information from Israel’s Prime Minister and social media.

Table 1 presents the characteristics and differences between the two groups in the two surveys. In the first survey, Arab participants were younger and more often born in Israel compared to Jewish participants. A larger proportion of Arab participants self-identified as traditional. In contrast, more Jewish participants reported chronic diseases, higher than average relative income, and receiving information in their native language. In the second survey, Arab participants were younger, had a higher level of education, and were more often born in Israel. A larger proportion of them self-identified as traditional or religious than the Jewish participants. In contrast, a larger proportion of Jewish participants reported receiving information in their native language and refraining from seeking healthcare.

**TABLE 1:** Distribution of study variables among Arab and Jewish participants in Israel in each of the surveys, 2020 (Israel, 2020). Inequalities in Trust Levels and Compliance With Physical Distancing During COVID-19 Outbreaks: Comparing the Arab Minority and Jewish Populations in Israel (Israel, 2020).

	The first survey (April–June 2020)				The second survey (October–November 2020)			
	Total	Jewish	Arab	*p*-value	Total	Jewish	Arab	*p*-value
	*n* = 613	*n* = 332 (54%)	*n* = 281 (46%)		*n* = 542	*n* = 319 (59%)	*n* = 223 (41%)	
Dependent variable								
Physical distancing compliance								
No	159 (25.9)	59 (17.8)	100 (35.6)	<0.001	153 (28.2)	78 (24.5)	75 (33.6)	0.021
Yes	454 (74.1)	273 (82.2)	181 (64.4)		388 (71.6)	240 (75.2)	148 (66.4)	
Independent variables								
Age								
(Mean + SD)	42.7 ± 15	44.9 ± 15.8	40 ± 13.5	<0.001	43.69 ± 14.42	43.62 ± 14.27	43.78 ± 14.66	0.75
Age groups								
18**–**29	127 (20.9)	53 (16.1)	74 (26.4)	<0.001	88 (16.2)	44 (13.8)	44 (19.7)	<0.01
30**–**39	150 (24.6)	86 (26.1)	64 (22.9)		146 (26.9)	101 (31.7)	45 (20.2)	
40**–**49	148 (24.3)	79 (24)	69 (24.6)		126 (23.2)	78 (24.5)	48 (21.5)	
50**–**59	108 (17.7)	53 (16.1)	55 (19.6)		95 (17.5)	43 (13.5)	52 (23.3)	
60+	76 (12.5)	58 (17.6)	18 (6.4)		87 (16.1)	53 (16.6)	34 (15.2)	
Gender								
Female	380 (62)	197 (59.3)	183 (65.1)	0.14	366 (67.5)	220 (69)	146 (65.5)	0.39
Male	233 (38)	135 (40.7)	98 (34.9)		176 (32.5)	99 (31)	77 (34.5)	
Education level								
Up to 12 years’ education	101 (16.4)	63 (19)	38 (13.5)	0.08	88 (16.2)	64 (20.1)	24 (10.8)	<0.01
Academic degree	512 (83.5)	269 (81)	243 (86.5)		454 (83.8)	255 (79.9)	199 (89.2)	
Marital status								
Married	404 (66)	230 (69.3)	174 (61.9)	0.05	376 (69.4)	219 (68.7)	157 (70.4)	0.5
Other	209 (34)	102 (30.7)	107 (38.1)		163 (30.1)	100 (31.3)	63 (28.3)	
Country of Birth								
Israel	553 (90)	278 (83.7)	280 (99.6)	<0.001	489 (90.2)	271 (85)	218 (97.8)	<0.001
Other	60 (10)	54 (16.3)	1 (0.4)		53 (9.8)	48 (15)	5 (2.2)	
Religiosity level								
Not Religious	399 (55.3)	235 (70.8)	104 (37.0)	<0.001	335 (61.8)	238 (74.6)	97 (43.5)	<0.001
Traditional	170 (27.7)	39 (11.7)	131 (46.6)		123 (22.7)	38 (11.9)	85 (38.1)	
Religious (or Haredi)	104 (17.0)	58 (17.5)	46 (16.4)		74 (13.7)	39 (12.2)	35 (15.7)	
Relative income								
Below average	78 (12.7)	39 (11.7)	39 (13.9)	0.04	79 (14.6)	48 (15)	31 (13.9)	0.19
Same as average	257 (41.9)	127 (38.3)	130 (46.3)		204 (37.6)	110 (34.5)	94 (42.2)	
Above average	278 (45.4)	166 (50)	112 (39.9)		259 (47.8)	161 (50.5)	98 (43.9)	
Chronic disease								
Yes	142 (23.6)	89 (26.8)	53 (18.9)	0.03	121 (22.3)	76 (23.8)	45 (20.2)	0.29
No	460 (76.4)	241 (72.6)	219 (77.9)		413 (76.2)	237 (74.3)	176 (78.9)	
State Anxiety								
Low	271 (47.8)	171 (55.5)	100 (38.6)	<0.001	200 (36.9)	126 (39.5)	171 (55.5)	0.134
High	296 (52.2)	137 (44.5)	159 (61.4)		342 (63.1)	193 (60.5)	137 (44.5)	
Smoking								
Yes	75 (12.2)	41 (12.3)	34 (12.1)	0.92	68 (12.5)	47 (14.7)	21 (9.4)	0.07
No	538 (87.8)	291 (87.7)	247 (87.9)		474 (87.5)	272 (85.3)	202 (90.6)	
Job loss due to lockdown								
Yes	193 (31.5)	105 (31.6)	88 (31.3)	0.86	186 (35.9)	108 (35.6)	78 (36.3)	0.94
No/other	361 (58.9)	197 (59.3)	164 (58.4)		291 (56.2)	170 (56.1)	121 (56.3)	
Not clear yet	59 (9.6)	30 (9)	29 (10.3)		41 (7.9)	25 (8.3)	16 (7.4)	
Information provided in the native language								
Slightly	150 (24.5)	54 (16.3)	96 (34.2)	<0.001	186 (34.3)	81 (25.4)	105 (47.1)	<0.001
Moderately	197 (32.1)	88 (26.5)	109 (38.8)		165 (30.4)	94 (29.5)	71 (31.8)	
Very much	266 (43.4)	190 (57.2)	76 (27.0)		191 (35.2)	144 (45.1)	47 (21.1)	
Refraining from seeking healthcare								
Yes					218 (40.2)	149 (46.7)	69 (30.9)	<0.001
No					324 (59.8)	170 (53.3)	154 (69.1)	
Do you know people who died from COVID-19?								
Yes					137 (25.8)	50 (15.8)	87 (40.5)	<0.001
No					395 (74.2)	267 (84.2)	128 (59.5)	

Note*.* data are *n*, (%) or mean (SD).

Table 2 presents the results of the univariate analyses. In the first survey, 82% of Jewish participants reported high levels of physical distancing compared with 64% of Arab participants. Among Jewish participants, those who reported a high level of trust (88%) also reported more compliance with physical distancing. Furthermore, those who did not suffer from chronic diseases (86%) and those who reported receiving information in their native language (87%) also reported more compliance with physical distancing. Among Arab participants, only those aged 60+ reported more compliance with physical distancing.

**TABLE 2:** Associations between study variables and physical distancing compliance among Arab and Jewish participants in Israel in each of the surveys, 2020 (Israel, 2020). Inequalities in Trust Levels and Compliance With Physical Distancing During COVID-19 Outbreaks: Comparing the Arab Minority and Jewish Populations in Israel (Israel, 2020).

Independent variables	The first survey (April–June 2020)			The second survey (October–November 2020)		
	Jewish	Arab	Total	Jewish	Arab	Total
	*n* = 332	*n* = 281	*n* = 613	*n* = 319	*n* = 223	*n* = 542
	(%) *n*	(%) *n*	(%) *n*	*n* (%)	*n* (%)	*n* (%)
Trust Level	*p* < 0.01	*p* = 0.11	*p* < 0.001	*p* = 0.05	*p* = 0.98	*p* = 0.07
Low	102 (74.5)	64 (39.5)	220 (66.9)	93 (69.9)	91 (66.4)	184 (68.1)
High	171 (87.7)	36 (30.3)	254 (80.9)	147 (79.5)	57 (66.3)	204 (75.3)
State Anxiety	*p* = 0.05	*p* = 0.57	*p* = 0.01	*p* = 0.98	*p* = 0.74	*p* = 0.93
Low	149 (87.1)	67 (67)	216 (79.7)	95 (75.4)	48 (64.9)	143 (71.5)
High	108 (78.8)	101 (63.5)	209 (70.9)	145 (75.5)	100 (67.1)	245 (71.8)
Age Groups	*p* = 0.51	*p* < 0.01	*p* < 0.01	*p* = 0.08	*p* = 0.02	*p* < 0.01
18–29	39 (73.6)	43 (58.1)	82 (64.6)	27 (61.4)	22 (50)	49 (55.7)
30–39	72 (83.7)	35 (54.7)	107 (71.3)	73 (72.3)	27 (60)	100 (68.5)
40–49	67 (84.4)	44 (63.8)	111 (75)	63 (81.8)	32 (66.7)	95 (76)
50–59	44 (83)	40 (72.7)	84 (77.8)	34 (79.1)	39 (75)	73 (76.8)
60+	51 (83.6)	19 (100)	70 (87.5)	43 (81.1)	28 (82.4)	71 (81.3)
Gender	*p* = 0.38	*p* = 0.20	*p* = 0.51	*p* = 0.29	*p* = 0.30	*p* = 0.29
Male	108 (80)	68 (69.4)	176 (75.5)	71 (71.7)	50 (64.9)	121 (68.8)
Female	165 (83.6)	113 (61.7)	278 (73.2)	169 (77.2)	98 (67.1)	267 (73.2)
Marital status	*p* = 0.23	*p* = 0.81	*p* = 0.19	*p* < 0.001	*p* = 0.23	*p* < 0.001
Married	80 (78.4)	68 (63.6)	306 (75.7)	61 (61)	38 (60.3)	287 (76.5)
Other	193 (83.9)	113 (64.9)	148 (70.8)	179 (82.1)	108 (68.8)	99 (60.7)
Religiosity level	*p* = 0.35	*p* = 0.39	*p* < 0.01	*p* = 0.66	*p* = 0.78	*p* = 0.80
Not Religious	192 (81.7)	70 (67.3)	262 (77.3)	178 (74.8)	65 (67)	243 (72.5)
Traditional	30 (76.9)	79 (60.3)	109 (64.1)	31 (81.6)	55 (64.7)	86 (69.9)
Religious (or Haredi)	51 (87.9)	32 (69.6)	83 (79.8)	29 (76.3)	25 (71.4)	54 (74)
Relative income	*p* = 0.60	*p* = 0.35	*p* = 0.10	*p* = 0.03	*p* = 0.98	*p* = 0.12
Below average	30 (76.9)	22 (56.4)	52 (66.7)	30 (63.8)	21 (67.7)	51 (65.4)
Same as average	104 (81.9)	82 (63.1)	186 (72.4)	79 (71.8)	62 (66)	141 (69.1)
Above average	139 (83.7)	77 (68.8)	216 (77.7)	131 (81.4)	65 (66.3)	196 (75.7)
Chronic disease	*p* < 0.01	*p* = 0.46	*p* = 0.36	*p* = 0.40	*p* = 0.15	*p* = 0.09
Yes	65 (73)	37 (69.8)	102 (71.8)	60 (78.9)	34 (75.6)	94 (77.7)
No	207 (85.9)	141 (64.4)	348 (75.7)	175 (74.2)	113 (64.2)	288 (69.9)
Smoking	*p* = 0.10	*p* = 0.97	*p* = 0.32	*p* = 0.85	*p* = 0.65	*p* = 0.95
Yes	30 (73.2)	22 (64.7)	52 (69.3)	36 (76.6)	13 (61.9)	49 (72.1)
No	243 (83.5)	159 (64.4)	402 (74.7)	204 (75.3)	135 (66.8)	339 (71.7)
Education level	*p* = 0.30	*p* = 0.37	*p* = 0.34	*p* < 0.01	*p* = 0.38	*p* = 0.01
Up to 12 years’ education	49 (77.8)	22 (57.9)	71 (70.3)	39 (61.9)	14 (58.3)	53 (60.9)
Academic degree	224 (83.3)	159 (65.4)	383 (74.8)	201 (78.8)	134 (67.3)	335 (73.8)
Job loss due to lockdown	*p* = 0.21	*p* = 0.56	*p* = 0.38	*p* = 0.63	*p* = 0.79	*p* = 0.92
Yes	168 (85.3)	106 (64.6)	274 (75.9)	131 (77.1)	77 (63.6)	208 (71.5)
No/Other	82 (78.1)	54 (61.4)	136 (70.5)	77 (72)	53 (67.9)	130 (70.3)
Not clear yet	23 (76.7)	21 (72.4)	44 (74.6)	19 (76)	11 (68.8)	30 (73.2)
Information provided in mother tongue language	*p* = 0.03	*p* = 0.61	*p* < 0.01	*p* = 0.82	*p* = 0.11	*p* = 0.17
Slightly	40 (74.1)	59 (61.5)	99 (66)	61 (75.3)	63 (60)	124 (66.7)
Moderately	68 (77.3)	74 (67.9)	142 (72.1)	73 (77.7)	49 (69)	122 (73.9)
Very much	165 (86.8)	48 (63.2)	213 (80.1)	106 (74.1)	36 (76.6)	142 (74.7)
Refraining from seeking healthcare				*p* = 0.66	*p* = 0.95	*p* = 0.94
Yes				110 (74.3)	46 (66.7)	156 (71.9)
No				130 (76.5)	102 (66.2)	232 (71.6)
Do you know people who died from COVID-19?				*p* = 0.13	*p* = 0.80	*p* = 0.92
Yes				42 (84)	57 (65.5)	99 (72.3)
No				197 (74.1)	86 (67.2)	283 (71.8)

Note. data are *n*, (%); *p*-values generated by chi-square tests.

In the second survey, 76% of Jewish participants reported high levels of physical distancing compared with 66% of Arab participants. Married participants (77%), elderly participants aged 60+ (81%), and participants with an academic degree (74%) also reported more compliance with physical distancing. Among Jewish participants, those who reported a higher than average relative income (81%), were unmarried (82%), and had an academic degree (79%) reported more compliance with physical distancing. Among Arab participants, there were no significant associations between trust and compliance with physical distancing in either survey. However, age was a significant factor, with elderly Arab participants aged 60+ reporting more compliance with physical distancing.

Table 3 shows the results of the multivariable logistic regressions. In the first survey, model 1 revealed more (vs. less) compliance with physical distancing among participants who reported having more trust (OR = 2.02, 95% CI = 1.37–2.97) than among those who reported having less trust. After adjusting for other variables (models 2 and 3), participants who reported a high level of trust were more likely to report compliance with physical distancing in both models (OR = 1.74, 95% CI = 1.13–2.67 and OR = 1.76, 95% CI = 1.08–2.57, respectively). Compared to Jewish participants, in the first survey Arab participants were less likely to report compliance with physical distancing in both models (OR = 0.52, 95% CI 0.32–0.84 and OR = 0.52, 95% CI 0.32–0.84, respectively). In addition, Jewish and Arab participants aged 60+ (OR = 4.06, 95% CI 1.57–10.47 and OR = 3.63, 95% CI 1.39–9.44, respectively) were more likely to report more (vs. less) compliance with physical distancing in their environment than younger participants (18–29 Y).

**TABLE 3:** Multivariable logistic regressions for physical distancing among the total sample in the first and second surveys in Israel, 2020 (Israel, 2020). Inequalities in Trust Levels and Compliance With Physical Distancing During COVID-19 Outbreaks: Comparing the Arab Minority and Jewish Populations in Israel (Israel, 2020).

	The first survey (April–June 2020) total (*n* = 613)						The second survey (October–November 2020) total (*n* = 542)					
	Model 1 (crude)		Model 2		Model 3		Model 1 (crude)		Model 2		Model 3	
	Crude OR (95% CI)	*p*	OR (95% CI)	*p*	OR (95% CI)	*p*	Crude OR (95% CI)	*p*	OR (95% CI)	*p*	OR (95% CI)	*p*
Trust levels												
Low	1		1		1		1		1		1	
High	2.02 (1.37–2.97)	<0.001	1.74 (1.13–2.67)	0.01	1.67 (1.08–2.57)	0.02	1.47 (1.00–2.15)	0.05	1.42 (0.93–1.16)	0.10	1.43 (0.93–2.17)	0.10
Age groups												
18**–**29			1		1				1		1	
30**–**39			1.25 (0.68–2.28)	0.47	1.21 (0.66–2.22)	0.53			1.37 (0.72–2.59)	0.33	1.41 (0.74–2.68)	0.30
40**–**49			1.43 (0.75–2.71)	0.28	1.31 (0.68–2.51)	0.42			1.83 (0.91–3.67)	0.09	1.81 (0.90–3.65)	0.10
50**–**59			2.06 (0.99–4.25)	0.05	1.96 (0.94–1.06)	0.07			2.36 (1.10–5.04)	0.03	2.37 (1.10–5.10)	0.03
60+			4.06 (1.57–10.47)	<0.01	3.63 (1.39–9.44)	<0.01			3.23 (1.46–7.16)	<0.01	3.20 (1.44–7.12)	<0.01
Ethnicity												
Jewish			1		1				1		1	
Arab			0.52 (0.32–0.84)	<0.01	0.52 (0.32–0.84)	<0.01			0.62 (0.39–0.98)	0.04	0.60 (0.68–0.97)	0.04
State anxiety												
Low			1		1				1		1	
High			0.78 (0.52–1.19)	0.25	0.78 (0.51–1.18)	0.24			1.21 (0.79–1.84)	0.38	1.25 (0.81–1.91)	0.31
Religiosity level												
Not Religious			1		1				1		1	
Traditional			0.83 (0.50–1.37)	0.46	0.83 (0.50–1.38)	0.48			1.12 (0.67–1.87)	0.68	1.02 (0.44–2.34)	0.97
Religious (or Haredi)			1.60 (0.85–2.99)	0.14	1.64 (0.87–3.08)	0.13			1.69 (0.87–3.26)	0.12	1.10 (0.44–2.78)	0.83
Relative income												
Below average			1		1				1		1	
Same as average			1.06 (0.57–1.98)	0.85	0.81 (0.31–2.09)	0.66			1.09 (0.59–1.99)	0.79	1.15 (0.62–2.14)	0.65
Above average			1.29 (0.68–2.44)	0.43	0.75 (0.30–1.92)	0.56			1.31 (0.71–2.39)	0.39	1.37 (0.74–2.55)	0.32
Marital status												
Unmarried			1		1				1		1	
Married			0.90 (0.55–1.48)	0.69	0.47 (0.16–1.41)	0.18			1.45 (0.90–2.32)	0.13	1.42 (0.88–2.29)	0.15
Education level												
Up to 12 years’ education			1		1				1		1	
Academic degree			1.19 (0.68–2.08)	0.55	1.12 (0.63–1.97)	0.71			1.37 (0.78–2.41)	0.27	1.36 (0.77–2.40)	0.29
Information provided in the native language												
Slightly			1		1				1		1	
Moderately			1.14 (0.68–1.90)	0.62	1.18 (0.70–1.96)	0.54			1.25 (0.76–2.06)	0.38	1.26 (0.66–2.42)	0.49
Very much			1.24 (0.73–2.13)	0.43	1.25 (0.73–2.15)	0.41			1.27 (0.75–2.12)	0.37	0.95 (0.51–1.78)	0.87
Interactions												
Unmarried * Below average Income					1							
Married * Average Income					1.83 (0.51–6.58)	0.36						
Married * Above Average Income					2.81 (0.77–10.30)	0.12						
Religiosity level: Not Religious * Information provided in the native language: Slightly											1	
Religiosity level: Traditional * Information provided in the native language: Moderately											0.82 (0.26–2.59)	0.73
Religiosity level: Traditional * Information provided in the native language: Very much											1.71 (0.52–5.89)	0.37
Religiosity level: Religious * Information provided in the native language: Moderately											1.34 (0.33–5.44)	0.69
Religiosity level: Religious * Information provided in the native language: Very much											1.75 (0.79–28.63)	0.09
-2 log-likelihood	625.46		582.73		580.14		624.57		591.33		586.86	

Note. OR refers to Odds Ratio.

In the second survey, model 2 showed that Arab participants were less likely to report compliance with physical distancing than Jewish participants (OR = 0.62, 95% CI 0.39–0.98). Moreover, model 2 and model 3 indicated that participants aged 60+(OR = 3.23, 95% CI 1.46–7.16 vs. OR = 3.2, 95% CI 1.44–7.12, respectively) and aged 50–59 (OR = 2.36, 95% CI 1.10–5.04 vs. OR = 2.37, 95% CI 1.10–5.10, respectively) were more likely to report compliance with physical distancing in their environment than younger participants aged 18–29.

To explore the factors contributing to compliance with physical distancing in each ethno-national group in the first and second surveys, we conducted separate multivariable analyses for Jews (Table 4) and Arabs (Table 5) separately. Significant associations emerged between trust in information sources and compliance with physical distancing among Jewish participants in the first and second surveys. Among Jewish participants in the first survey, model 1 shows that those who reported a high (vs. low) level of trust (OR = 2.52, 95% CI 1.36–4.66) were more likely to report compliance with physical distancing. After adjusting for other independent variables in models 2 and 3, Jewish participants who reported a high (vs. low) level of trust (OR = 2.51, 95% CI 1.29–4.90 and OR = 2.45, 95% CI 1.24–4.84, respectively) were more likely to report more (vs. less) compliance with physical distancing. In the second survey, model 1 shows that Jewish participants who reported a high (vs. low) level of trust (OR = 1.76, 95% CI 1.05–4.96) were more likely to report compliance with physical distancing. After adjusting for other independent variables in models 2 and 3, married (vs. unmarried) Jewish participants (OR = 2.17, 95% CI 1.16–4.05 and OR = 2.14, 95% CI 1.13–4.04, respectively) reported more (vs. less) compliance with physical distancing.

**TABLE 4:** Multivariable logistic regressions for physical distancing among Jewish participants in the first and second survey in Israel, 2020 (Israel, 2020). Inequalities in Trust Levels and Compliance With Physical Distancing During COVID-19 Outbreaks: Comparing the Arab Minority and Jewish Populations in Israel (Israel, 2020).

	The first survey (April–June 2020) total (*n* = 332)						The second survey (October–November 2020) total (*n* = 319)					
	Model 1 (crude)		Model 2		Model 3		Model 1 (crude)		Model 2		Model 3	
	Crude OR (95% CI)	*p*	OR (95% CI)	*p*	OR (95% CI)	*p*	Crude OR (95% CI)	*p*	OR (95% CI)	*p*	OR (95% CI)	*p*
Trust levels												
Low	1		1		1		1		1		1	
High	2.52 (1.36–4.66)	<0.01	2.51 (1.29–4.90)	<0.01	2.45 (1.24–4.84)	0.01	1.76 (1.05–2.96)	0.03	1.76 (0.99–3.15)	0.06	1.77 (0.99–3.16)	0.06
Age groups												
18**–**29			1		1				1		1	
30**–**39			1.67 (0.57–4.83)	0.35	1.82 (0.60–5.45)	0.29			0.90 (0.36–2.24)	0.82	0.95 (0.37–2.42)	0.91
40**–**49			2.80 (0.87–9.05)	0.09	2.89 (0.86–9.65)	0.09			1.27 (0.46–3.49)	0.65	1.32 (0.47–3.69)	0.60
50**–**59			1.62 (0.49–5.35)	0.43	1.81 (0.53–6.20)	0.35			1.35 (0.42–4.27)	0.61	1.39 (0.43–4.49)	0.59
60+			3.33 (0.93–11.87)	0.06	3.66 (0.99–13.52)	0.05			1.61 (0.55–4.69)	0.38	1.72 (0.58–5.07)	0.32
State anxiety												
Low			1		1				1		1	
High			0.63 (0.33–1.21)	0.17	0.61 (0.32–1.18)	0.15			1.16 (0.65–2.08)	0.62	1.22 (0.68–2.20)	0.50
Religiosity level												
Not religious			1		1				1		1	
Traditional			0.60 (0.23–1.56)	0.29	0.52 (0.21–1.54)	0.27			1.45 (0.57–3.73)	0.44	591,164,987.77 (NA)	1.00
Religious (or Haredi)			1.60 (0.58–4.44)	0.37	1.78 (0.61–5.15)	0.29			1.22 (0.47–3.17)	0.68	0.92 (0.21–3.90)	0.91
Relative Income												
Below average			1		1				1		1	
Same as average			0.76 (0.27–2.19)	0.62	0.22 (0.04–1.24)	0.09			1.09 (0.47–2.52)	0.84	1.11 (0.47–2.60)	0.81
Above average			0.98 (0.33–2.90)	0.97	0.64 (0.11–3.75)	0.62			1.68 (0.72–3.89)	0.23	1.73 (0.73–4.09)	0.21
Marital status												
Unmarried			1		1				1		1	
Married			1.01 (0.48–2.14)	0.98	0.28 (0.04–1.96)	0.20			2.17 (1.16–4.05)	0.02	2.14 (1.13–4.04)	0.02
Education level												
Up to 12 years’ education			1		1				1		1	
Academic degree			0.80 (0.32–1.99)	0.63	0.71 (0.27–1.81)	0.47			1.42 (0.68–2.96)	0.35	1.36 (0.64–2.87)	0.43
Information provided in the native language												
Slightly			1		1				1		1	
Moderately			1.14 (0.46–2.82)	0.77	1.26 (0.50–3.13)	0.63			1.14 (0.54–2.41)	0.74	1.24 (0.52–2.92)	0.63
Very much			1.86 (0.81–4.29)	0.14	1.87 (0.80–4.36)	0.15			0.88 (0.43–1.77)	0.71	0.85 (0.38–1.85)	0.68
Interactions												
Unmarried * Below average Income					1							
Married * Average Income					8.20 (0.91–74.09)	0.06						
Married * Above Average Income					2.44 (0.26–23.25)	0.44						
Religiosity level: Not Religious * Information provided in the native language: Slightly											1	
Religiosity level: Traditional * Information provided in the native language: Moderately											0.00 (NA)	1.00
Religiosity level: Traditional * Information provided in the native language: Very much											0.00 (NA)	1.00
Religiosity level: Religious * Information provided in the native language: Moderately											1.26 (0.16–9.98)	0.83
Religiosity level: Religious * Information provided in the native language: Very much											2.17 (0.24–19.23)	0.49
-2 log-likelihood	267.61		254.95		250.19		342.97		322.06		319.28	

Note. OR refers to Odds Ratio.

**TABLE 5:** Multivariable logistic regressions for physical distancing among Arab participants in the first and second survey in Israel, 2020 (Israel, 2020). Inequalities in Trust Levels and Compliance With Physical Distancing During COVID-19 Outbreaks: Comparing the Arab Minority and Jewish Populations in Israel (Israel, 2020).

	The first survey (April–June 2020) total (*n* = 281)						The second survey (October–November 2020) total (*n* = 238)					
			Model 2		Model 3		Model 1 (crude)		Model 2		Model 3	
	Crude OR (95% CI)	*p*	OR (95% CI)	*p*	OR (95% CI)	*p*	Crude OR (95% CI)	*p*	OR (95% CI)	*p*	OR (95% CI)	*p*
Trust levels												
Low	1		1		1		1		1		1	
High	1.39 (0.82–2.33)	0.22	1.36 (0.75–2.46)	0.31	1.23 (0.67–2.25)	0.50	0.99 (0.55–1.77)	0.97	1.05 (0.54–2.04)	0.88	1.03 (0.52–2.01)	0.94
Age group												
18**–**29			1		1				1		1	
30**–**39			1.17 (0.53–2.58)	0.70	1.15 (0.52–2.56)	0.72			2.27 (0.86–5.95)	0.10	2.33 (0.87–6.26)	0.09
40**–**49			1.20 (0.53–2.76)	0.66	1.15 (0.49–2.70)	0.75			2.98 (1.05–8.40)	0.04	3.03 (1.06–8.64)	0.04
50**–**59			3.14 (1.19–8.28)	0.02	3.11 (1.17–8.28)	0.02			4.92 (1.64–14.79)	<0.01	5.27 (1.73–16.04)	<0.01
60+			1,479,207,015.09 (NA)	1.00	1,349,411,323.85 (NA)	1.00			8.62 (2.43–30.65)	<0.001	8.77 (2.43–31.64)	<0.01
State anxiety												
Low			1		1				1		1	
High			0.78 (0.44–1.38)	0.39	0.74 (0.41–1.33)	0.32			1.32 (0.67–2.58)	0.42	1.39 (0.70–2.76)	0.35
Religiosity level												
Not religious			1		1				1		1	
Traditional			0.99 (0.53–1.83)	0.96	0.94 (0.50–1.75)	0.84			1.03 (0.52–2.04)	0.93	1.09 (0.41–2.84)	0.87
Religious (or Haredi)			1.76 (0.74–4.19)	0.20	1.63 (0.68–3.94)	0.28			2.33 (0.87–6.19)	0.09	1.56 (0.43–5.59)	0.50
Relative income												
Below average			1		1				1		1	
Same as average			1.33 (0.57–3.11)	0.52	1.88 (0.54–6.58)	0.32			0.94 (0.36–2.49)	0.91	0.96 (0.35–2.62)	0.94
Above average			1.51 (0.63–3.58)	0.35	0.90 (0.26–3.04)	0.86			0.83 (0.32–2.19)	0.71	0.79 (0.29–2.14)	0.65
Marital status												
Unmarried			1		1				1		1	
Married			0.72 (0.36–1.44)	0.36	0.59 (0.13–2.56)	0.48			0.79 (0.36–1.76)	0.57	0.77 (0.34–1.75)	0.54
Education level												
Up to 12 years’ education			1		1				1		1	
Academic degree			1.82 (0.82–4.05)	0.14	1.66 (0.74–3.72)	0.22			1.21 (0.44–3.30)	0.71	1.38 (0.48–3.97)	0.55
Information provided in the native language												
Slightly			1		1				1		1	
Moderately			1.32 (0.69–2.52)	0.41	1.41 (0.72–2.73)	0.31			1.41 (0.69–2.90)	0.35	1.66 (0.54–5.03)	0.37
Very much			0.87 (0.41–1.84)	0.71	0.98 (0.45–2.11)	0.95			2.21 (0.91–5.38)	0.08	1.14 (0.30–4.24)	0.85
Interactions												
Unmarried * Below average Income					1							
Married * Average Income					0.66 (0.12–3.60)	0.63						
Married * Above Average Income					2.83 (0.49–16.25)	0.24						
Religiosity level: Not Religious * Information provided in the native language: Slightly											1	
Religiosity level: Traditional * Information provided in the native language: Moderately											0.65 (0.14–3.02)	0.58
Religiosity level: Traditional * Information provided in the native language: Very much											1.90 (0.31–11.67)	0.49
Religiosity level: Religious * Information provided in the native language: Moderately											1.10 (0.14–8.47)	0.93
Religiosity level: Religious * Information provided in the native language: Very much											1,243,531,138.95 (NA)	1.00
-2 log-likelihood	334.30		305.93		300.37		275.78		254.83		249.37	

Note. OR refers to Odds Ratio.

Among Arab participants, no significant association emerged in either survey between trust and compliance with physical distancing (Table 5). In the first survey, models 2 and 3 showed that compared to young participants aged 18–29, Arab participants aged 50–59 were more likely to report more (vs. less) compliance with physical distancing in their environment (OR = 3.14, 95% CI 1.19–8.28 and OR = 3.11, 95% CI 1.17–8.28, respectively). In the second survey, models 2 and 3 indicated that compared to young participants aged 18–29, Arab participants aged 60+ (OR = 8.62, 95% CI 2.43–30.65 and OR = 8.77, 95% CI 2.43–31.64, respectively), and those 50–59 (OR = 4.92, 95% CI 1.64–14.79 and OR = 5.27, 95% CI 1.73–16.04, respectively) were more likely to report more (vs. less) compliance with physical distancing in their environment.

## Discussion

This study contributes to the public health literature on associations between trust and compliance with public directives. Here, we focused on compliance with physical distancing directives in two ethno-national groups during COVID-19 lockdowns. It is the first study to compare these associations in the Jewish majority and Arab minority in Israel.

A main finding was that trust in directives was significantly associated with compliance with physical distancing among the total sample in the first survey conducted after the first lockdown. Participants who reported a high (vs. low) level of trust were more likely to report more (vs. less) compliance with physical distancing (OR = 2.02, 95% CI 1.37–2.97) even after adjusting for the independent variables. This finding accords with the results of an Israeli study suggesting that individuals with generally low levels of trust may be less likely to comply with COVID-19 guidelines (32). Studies conducted in Europe and the US also support our findings (11, 33). For example, a study conducted in the US reported greater compliance with physical distancing among people who had more trust in science (33). However, cultural orientation within different countries may influence the meaning and implications of trust and compliance with guidelines (34). Thus, this comparison should be interpreted cautiously. Note, too, that our study’s measurements of trust and compliance with physical distancing were different from those used in the other studies (33, 35–37). We measured compliance with physical distancing by asking participants about compliance in their environment rather than individual compliance. We did so to avoid social desirability bias (38) because non-compliance with physical distancing guidelines in Israel at that time was considered a violation of the law and public health regulations.

Our results indicate that, while a significant association between trust in information sources and compliance with physical distancing emerged in both surveys among the Jewish participants, this association was not significant in either survey among the Arab participants. One explanation might be that Arabs generally have less trust in the government and related institutions than their Jewish counterparts (39). Feelings of alienation among the Arab population in Israel due to discriminatory policies (22, 40) might also impact trust in directives related to COVID-19 (41). There were significant differences in compliance with physical distancing between the groups in both surveys. Compared to Jewish participants, Arabs reported less compliance with physical distancing, both in the first survey (OR = 0.52, 95% CI 0.32–0.84) and in the second one (OR = 0.62, 95% CI 0.39–0.98). One explanation for these differences might be related to the fact that the first survey was conducted during Ramadan 2020, the holy month for Muslims when it is customary to gather for festive meals, visit families and friends, engage in spiritual practices in enclosed spaces such as mosques and use common cleaning facilities before prayers, which may impede physical distancing. Furthermore, at the beginning of the COVID-19 outbreak, most official statements were not issued in Arabic and much of the information that was eventually delivered in Arabic was delayed. In the first survey, 34% of Arab participants reported receiving less information in their native language (Arabic) compared to 16% of Jewish participants. Thus, it is probable that physical distancing guidelines were less accessible for the Arab minority.

Although this was not a longitudinal study, we still found a decrease in compliance with physical distancing among the total sample between the first (74%) and second surveys (72%) (Figure 1). These results accord with a recent UK-based study, also conducted during the pandemic, indicating that about one in seven participants reported a decline in compliance with physical distancing (42). Additionally, previous research conducted in Italy has suggested that more trust in political and administrative institutions increased the level of compliance with public health regulations, while anxiety about the future and fatigue explain why less trust in governmental organizations reduced the adoption of protective behaviors (43). The drop in compliance in the second wave may be indicative of behavioral fatigue (42, 44) and future anxiety (43). Explanations for the decline in compliance found in our study include the loosening of COVID-19 restrictions, lack of strict enforcement of regulations and an erosion of trust in the Israeli public. Notably, this erosion was evident in both groups. According to our results, there was an increase between the first and second surveys in the rate of participants reporting a loss of employment (31.5% and 35.9%, respectively). Thus, the eroded trust might be related to increasing employment loss due to lockdowns and the absence of government financial support (45). In addition, the instructions changed over time, and there was a feeling of politicizing the pandemic. We also considered the role of several socio-demographic factors. One factor that could prove significant is the influence of age in the slight increase in compliance with physical distancing among Arab participants that took place between the first and second surveys. After adjusting for the independent variables, Arab participants aged 50–59 were more likely to report more (vs. less) compliance with physical distancing in their environment compared to young participants aged 18–29. In the second survey, participants from all the age groups (except the 30–39 age group) were more likely to report more (vs. less) compliance with physical distancing in their environment compared to young participants aged 18–29. We also documented that the older the person, the more likely s/he indicated compliance with physical distancing in his/her environment. One explanation for this finding is the strong sense of community, solidarity and social cohesion in the Arab population in Israel (46). Given that being older is an independent risk factor for COVID-19 mortality (47), people probably tended to take care of each other much more.

Turning to other characteristics of the participants who reported more compliance with physical distancing, our results are consistent with previous research. A study in the US conducted during the COVID-19 pandemic (48) indicated that elderly respondents were less likely to engage in risky behaviors. Similarly, our findings showed that elderly participants aged 60+ from both groups were more likely to comply with physical distancing than younger participants. Such compliance was probably rooted in their recognition of being at greater risk from COVID-19 (49).

Overall, our results emphasize that trust in information sources is an important determinant of citizens’ compliance with physical distancing guidelines in the early stages of the pandemic. It is also important to maintain a high level of trust over time, especially when an emergency like COVID-19 lasts longer than expected. In addition, policymakers should note the differences we found between the two ethno-national groups, as the consistently lower level of trust of the minority group might have significant consequences in future times of crisis when the government will again need all groups to comply with health directives.

### Strengths and Limitations

In addition to our contributions, our study has a number of limitations. In rapidly evolving situations like COVID-19, conducting research through online surveys and utilizing a convenience sample is a useful source of knowledge. Yet, this strategy might be a potential source of selection bias and could limit generalization of the results. We tried to reach different population groups by distributing the questionnaire’s hyperlink through different media channels and social media groups. In addition, given that we used an online data collection method, we obtained information only from those who had access to the Internet and online social media forums, rather than from a larger or harder-to-reach population. Furthermore, although using an indirect question about compliance with physical distancing during the COVID-19 pandemic might have influenced social desirability, it was not clear to what extent. Finally, we used cross-sectional surveys that do not imply causality but this was the optimal method during the COVID-19 outbreak. Still, the results provide valuable insights into variations in trust in information sources and compliance with physical distancing between two different groups in Israel.

### Conclusion

To the best of our knowledge, this is the first study to examine the association between trust and compliance with public distancing in Israel during COVID-19. Our findings indicate that momentum is important in building and maintaining public trust and compliance during pandemics. Policymakers should note the lack of trust among the Arab minority, which warrants further research and interventions.
